# Biphasic Effects of α-Asarone on Immobility in the Tail Suspension Test: Evidence for the Involvement of the Noradrenergic and Serotonergic Systems in Its Antidepressant-Like Activity

**DOI:** 10.3389/fphar.2016.00072

**Published:** 2016-03-30

**Authors:** Ranjithkumar Chellian, Vijayapandi Pandy, Zahurin Mohamed

**Affiliations:** Department of Pharmacology, Faculty of Medicine, University of MalayaKuala Lumpur, Malaysia

**Keywords:** depression, AMPT, PCPA, prazosin, yohimbine, WAY100635

## Abstract

Alpha (α)-asarone is one of the main psychoactive compounds, present in *Acorus* species. Evidence suggests that the α-asarone possess an antidepressant-like activity in mice. However, the exact dose-dependent effect of α-asarone and mechanism(s) involved in the antidepressant-like activity are not clear. The present study aimed to investigate the dose-dependent effect of α-asarone and the underlining mechanism(s) involved in the antidepressant-like activity of α-asarone in the mouse model of tail suspension test (TST). In this study, the acute effect of α-asarone *per se* at different doses (10–100 mg/kg, i.p.) on immobility in the TST was studied. Additionally, the possible mechanism(s) involved in the antidepressant-like effect of α-asarone was studied using its interaction with noradrenergic and serotonergic neuromodulators in the TST. The present results reveal that the acute treatment of α-asarone elicited biphasic responses on immobility such that the duration of the immobility time is significantly reduced at lower doses (15 and 20 mg/kg, i.p.) but increased at higher doses (50 and 100 mg/kg, i.p.) in the TST. Besides, α-asarone at higher doses (50 and 100 mg/kg, i.p.) significantly decreased the spontaneous locomotor activity. Moreover, pretreatment of mice with noradrenergic neuromodulators such as AMPT (100 mg/kg, i.p., a catecholamine synthesis inhibitor), prazosin (1 mg/kg, i.p., an α_1_-adrenoceptor antagonist), yohimbine (1 mg/kg, i.p., an α_2_-adrenoceptor antagonist) and with serotonergic neuromodulators such as PCPA (100 mg/kg, i.p., once daily for four consecutive days, a serotonin synthesis inhibitor,) and WAY100635 (0.1 mg/kg, s.c., a selective 5-HT_1A_ receptor antagonist) significantly reversed the anti-immobility effect of α-asarone (20 mg/kg, i.p.). Taken together, our results suggest that the acute treatment with α-asarone elicited biphasic actions in the TST in which antidepressant-like effect was seen at relatively lower doses (15 and 20 mg/kg, i.p.) and depressive-like activity at relatively higher doses (50 and 100 mg/kg, i.p.). Furthermore, it has been revealed that the antidepressant-like effect of α-asarone could be mediated through both noradrenergic (α_1_ and α_2_ adrenoceptors) and serotonergic (particularly, 5-HT_1A_ receptors) systems.

## Introduction

**FIGURE 1:**
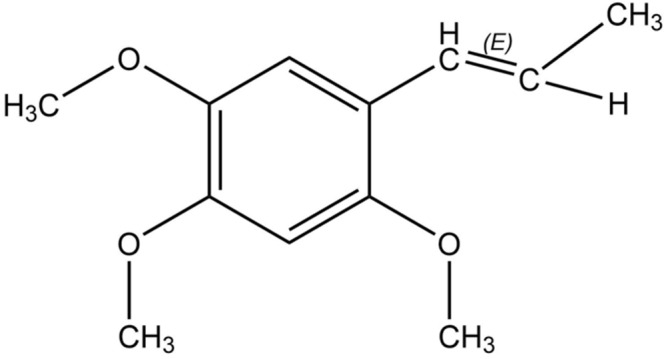
**Chemical structure of α-asarone (1, 2, 4-trimethoxy-5-[(E)-pro-1-enyl] benzene)**.

Alpha (α)-asarone [1,2,4-trimethoxy-5-[(E)-pro-1-enyl] benzene; Pubchem CID: 636822; **Figure 1**), is one of the main pharmacologically active compounds present in *Acorus calamus* Linn (Acoraceae), *Acorus tatarinowii* Schott (Acoraceae), and *Acorus gramineus* Solander (Acoraceae; Rajput et al., 2014). The various neuropharmacological activities of α-asarone in numerous preclinical studies were reported in the literature including anticonvulsant (Huang et al., 2013), neuroprotective (Limon et al., 2009), anxiolytic (Liu et al., 2012), and nootropic effects (Limon et al., 2009; Kumar et al., 2012). Recently, the antidepressant-like effect of an essential oil from *Acorus tatarinowii* Schott have been reported in well-validated animal models of depression such as TST and forced swim test (FST). In the same study, the acute treatment of α-asarone at lower doses (10 and 20 mg/kg, i.p.) showed an antidepressant-like activity in both established mouse models (Han et al., 2013). Besides that, other reports claimed that α-asarone possess CNS depressant-like effect whereby mice treated with α-asarone at higher doses (≥50 mg/kg, i.p.) affected locomotor activity and potentiated the pentobarbitone-induced sleeping time, a test that is used for the screening of potential CNS depressants (Menon and Dandiya, 1967; Pages et al., 2010; Liu et al., 2012). Similarly, CNS depressant-like activity of roots, rhizome and leaf extracts of *Acorus calamus* have been reported (Motley, 1994; Pandy et al., 2009). In our previous study, *Acorus calamus* leaf extracts significantly increased the immobility time in FST, and diazepam-induced sleeping time and significantly reduced the spontaneous locomotor activity without affecting motor coordination (Pandy et al., 2009). Conversely, antidepressant-like activities of methanolic extract of rhizomes and leaves of *Acorus calamus* in FST and TST have been reported in other studies (Pawar Vinod et al., 2012; Pushpa et al., 2013). These seemingly contradictory reports led us to try and find answers by conducting further studies to determine dose-dependent effect of α-asarone, the active phytoconstituent of *Acorus* species, at doses that are beyond those that have been reported, which is more than 20 mg/kg in the TST. Additionally, the underlying mechanism(s) involved in the antidepressant-like activity of α-asarone was examined using its interaction with noradrenergic neuromodulators such as AMPT, prazosin, and yohimbine and serotonergic neuromodulators PCPA and WAY100635 in the TST.

## Materials and Methods

### Animals

Adult male, ICR mice (Institute for Cancer Research) of age 8–10 weeks, bred and supplied by the Animal Experimental Unit (AEU, Faculty of Medicine, University of Malaya, Kuala Lumpur) were used in all our experiments. The mice were housed (four mice per cage) in an individually ventilated cage at the Satellite Animal Facility (SAF), Department of Pharmacology, Faculty of Medicine, University of Malaya, Kuala Lumpur, and acclimatized for a week in a controlled environment [22 ± 2°C, 50–70% humidity and 12 h light/dark (lights on at 7.00 am)] with food and water available *ad libitum*. AEU and SAF have been accredited by the Association for Assessment and Accreditation of Laboratory Animal Care International (AAALAC). All experimental protocols adhered to the guidelines of the National Research Council of the National Academies of the USA (“Guide for the Care and Use of Laboratory Animals,” Eighth Edition; Garber et al., 2011) and were assessed and approved by the Faculty of Medicine-Institutional Animal Care and Use Committee (FOM-IACUC), University of Malaya (Ethics Approval no: 2014-10-14/PHAR/R/VP). The behavioral experiments were performed during the light cycle between 10.00 am and 6.00 pm. All efforts were made to minimize suffering in the mice, and to reduce the number of mice used in the experiments.

### Drugs and Treatment

The following drugs were used: α-asarone (Lot # S18779V; Purity 98% w/w), Tween 80 (polyethylene sorbitan monooleate; Lot # MKBP0682V; Purity ≥ 99% v/v), α-methyl-*p*-tyrosine (AMPT; Lot # STBD4408V; Purity 98% w/w), and 4-chloro-D-L-phenylalanine methyl ester hydrochloride (PCPA or Fenclonine; Lot # SHBD9164V; Purity 97% w/w) ± 8-hydroxy-2-dipropylamino tetralin hydrobromide (8-OH-DPAT; Lot # 053M4102V; Purity ≥ 98% w/w), prazosin hydrochloride (Lot # 129K1137V; Purity ≥ 99% w/w) and yohimbine hydrochloride (Lot # 13CBM8231V; Purity ≥ 98% w/w; purchased from Sigma-Aldrich, St. Louis, MO, USA); bupropion hydrochloride (Lot #2596608; Purity ≥ 99.5% w/w) and fluoxetine hydrochloride (Lot #2597489; Purity 99.8% w/w; obtained from LKT laboratories, Inc., St. Paul, MN, USA); *N*-[2-[4-(2-methoxyphenyl) piperazin-1-yl] ethyl]- *N*-pyridin-2-ylcyclohexanecarcoxamide hydrochloride (WAY100635; Lot # 6-GJF-12-1; Purity 98% w/w; purchased from Toronto Research Chemicals, Inc., Toronto, ON, Canada). The α-asarone was suspended in 5% v/v Tween 80 prepared in normal saline. Bupropion, fluoxetine, PCPA, WAY100635, 8-OH-DPAT, prazosin and yohimbine were dissolved in normal saline and AMPT was suspended in 10% v/v Tween 80 prepared in normal saline. All the drugs were administered i.p., whereas WAY100635 was administered s.c. on a constant dose volume of 10 mL/kg body weight of mice. The mice in the control group received the appropriate vehicle used in this study. The administration schedule and dose of drugs used in this study was chosen as reported in the published literature and standardized in our laboratory.

### Behavioral Procedures

#### Tail Suspension Test

The TST was performed for 6 min as described previously (Steru et al., 1985). Briefly, both acoustically and visually isolated mice were suspended 25-cm above the floor by adhesive tape placed approximately 1 cm from the tip of the tail. Mice were considered immobile when they hung passively and completely motionless. The motionless hanging posture represents depression-like behavior of the animals. The experiment was recorded and monitored using a Logitech webcam (C270) connected to a personal computer and the immobility time in second was measured using a digital stop-watch during the 6 min test by an experienced observer (blinded to the experiment). Antidepressants decrease the immobility time in the TST (Steru et al., 1985).

#### Horizontal Wire Test

The experiment was performed as described previously (Jung et al., 2013) with slight modifications. The mouse was lifted by the tail and the forepaws were allowed to grasp the center of the horizontal metallic wire (2 mm diameter, 70 cm long) suspended in the air about 40 cm from the surface of the table and then the tail was released to let the mouse to hang with its forelimb. The ability of the mouse to actively grasp the wire within the first 10 s (grasping reflex) and hang on, or climb up within 20 s test was measured. The mouse which tends to grasp, hang or climb-up, was considered as a normal motor coordination. On the other hand, mouse which failed to grasp or fall off from the wire within 20 s was considered as mouse with impaired motor coordination. The data are expressed as % of mice with normal motor coordination.

### Spontaneous Locomotor Activity

The spontaneous locomotor activity was assessed using actimeter (Model: ACT-01, Orchid’s Scientific, Nasik, India) fabricated with clear square Plexiglas arena (50 cm × 50 cm), equipped with 32-infrared sensors. The mouse was placed in the center of the arena and the locomotor activity was measured for the duration of 10 min. The data are expressed as the total light beam interruptions (locomotor counts). The floor of the apparatus was cleaned with 20% v/v ethanol between tests.

### Experimental Design

#### Effect of α-Asarone *per se* in the TST

The mice were divided into eight groups (*N* = 10). Thirty minutes after acute treatment with the vehicle (5% v/v Tween 80) or with bupropion (20 mg/kg, i.p.) as a positive control or the test compound α-asarone (10, 15, 20, 30, 50, and 100 mg/kg, i.p.), the immobility time in seconds was measured in the TST.

#### Effect of α-Asarone in the Horizontal Wire Test

The mice were assessed for motor coordination in the horizontal wire test. Briefly, seven groups (*N* = 9–10) were treated with an acute dose of vehicle (5% v/v Tween 80) or with α-asarone (10, 15, 20, 30, 50, and 100 mg/kg, i.p.). Thirty minutes after acute treatment with vehicle or α-asarone, the horizontal wire test was performed.

#### Effect of α-Asarone in the Spontaneous Locomotor Activity

The mice were divided into seven groups (*N* = 9–10). Thirty minutes after acute treatment with vehicle (5% v/v Tween 80) or α-asarone (10, 15, 20, 30, 50, and 100 mg/kg, i.p.), the spontaneous locomotor activity was assessed for 10 min in actimeter.

### Investigation of the Involvement of Noradrenergic System in the Antidepressant-Like Effect of α-Asarone

To investigate the involvement of the noradrenergic system in the antidepressant-like activity of α-asarone, the mice were pretreated with saline, or AMPT (100 mg/kg, i.p., a catecholamine synthesis inhibitor). Four hours after AMPT administration, mice were treated with either vehicle (5% v/v Tween 80) or bupropion (20 mg/kg, i.p.) or α-asarone (20 mg/kg, i.p.). Thirty minutes after vehicle or drug treatment, TST was performed (Machado et al., 2008; Kwon et al., 2010). In another study, the mice were pretreated with saline or prazosin (1 mg/kg, i.p., an α_1_-adrenoceptor antagonist) or yohimbine (1 mg/kg, i.p., an α_2_-adrenoceptor antagonist). Thirty minutes after administration of saline or prazosin, or yohimbine, mice were treated with either vehicle (5% v/v Tween 80) or α-asarone (20 mg/kg, i.p.). Thirty minutes after vehicle or α-asarone treatment, the immobility time in seconds was measured in the TST (Colla et al., 2012; Zeni et al., 2013).

### Investigation of the Involvement of Serotonergic System in the Antidepressant-Like Effect of α-Asarone

To assess the involvement of serotonergic system in the antidepressant-like activity of α-asarone, the mice were pretreated with saline, or PCPA (100 mg/kg, i.p., a serotonin synthesis inhibitor, once daily for four consecutive days). On day 5 (24 h after last PCPA treatment), mice received either vehicle (5% v/v Tween 80) or fluoxetine (30 mg/kg, i.p.) or α-asarone (20 mg/kg, i.p.) 30 min prior to TST (Machado et al., 2009; Kwon et al., 2010). In another study, the mice were pretreated with either saline or WAY100635 (0.1 mg/kg, s.c., a selective 5-HT_1A_ receptor antagonist). Thirty minutes after saline or WAY100635 treatment, the mice were administered with either vehicle (5% v/v Tween 80) or 8-OH-DPAT (1 mg/kg, i.p., a selective 5-HT_1A_ receptor agonist) or α-asarone (20 mg/kg, i.p.). The immobility time in seconds was measured after 30 min of vehicle or 8-OH-DPAT or α-asarone (Talbot et al., 2010; Colla et al., 2012).

### Statistical Analysis

Values are expressed as mean ± SEM. The behavioral tests were analyzed by one-way ANOVA followed by *post hoc* Dunnett’s multiple comparison test or two-way analysis of variance (two-way ANOVA) followed by *post hoc* Bonferroni test using Graphpad prism 5.03 (Graphpad Software, Inc., USA). Statistical significance was set at *p* < 0.05.

## Results

### Effect of α-Asarone *per se* in the TST

**FIGURE 2:**
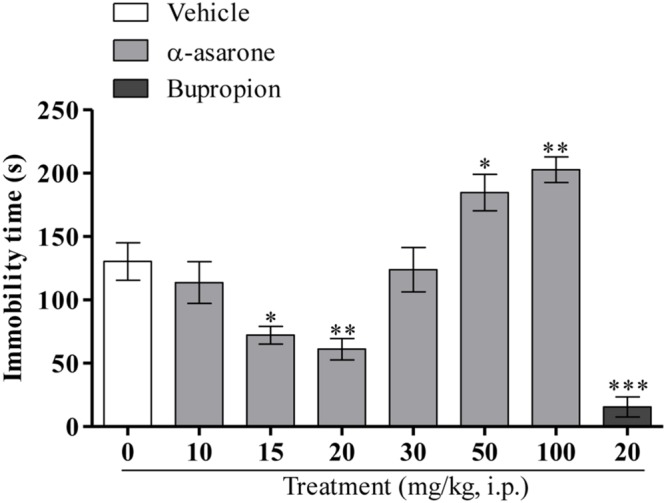
**Tail suspension test.** Effect of the acute treatment of α-asarone (10, 15, 20, 30, 50, and 100 mg/kg, i.p.) *per se* and bupropion (20 mg/kg, i.p.) on the immobility time of mice in the TST. Data are expressed as mean ± SEM (*n* = 10). The statistical difference between vehicle and α-asarone or bupropion was analyzed using one-way ANOVA followed by *post hoc* Dunnett’s multiple comparison test. ^∗^*p* < 0.05, ^∗∗^*p* < 0.01, and ^∗∗∗^*p* < 0.001 as compared with vehicle-control group.

As shown in (**Figure 2**), one-way ANOVA results revealed that the acute treatment of α-asarone at relatively lower doses (15 and 20 mg/kg, i.p.) and the reference antidepressant, bupropion (20 mg/kg, i.p.) significantly reduced the immobility time in the TST. In contrast, α-asarone at relatively higher doses (50 and 100 mg/kg, i.p.) significantly increased the immobility time in the TST as compared with vehicle control [*F*_(6,62)_ = 15.30, *p* < 0.0001].

### Effect of α-Asarone *per se* in the Horizontal Wire Test

The results of the horizontal wire test revealed that α-asarone (10, 15, 20, 30, 50, and 100 mg/kg, i.p.) did not alter the normal motor coordination that is, it did not affect the grasping reflex, hanging or climbing behavior (data not shown).

### Effect of α-Asarone *per se* on the Spontaneous Locomotor Activity

**FIGURE 3:**
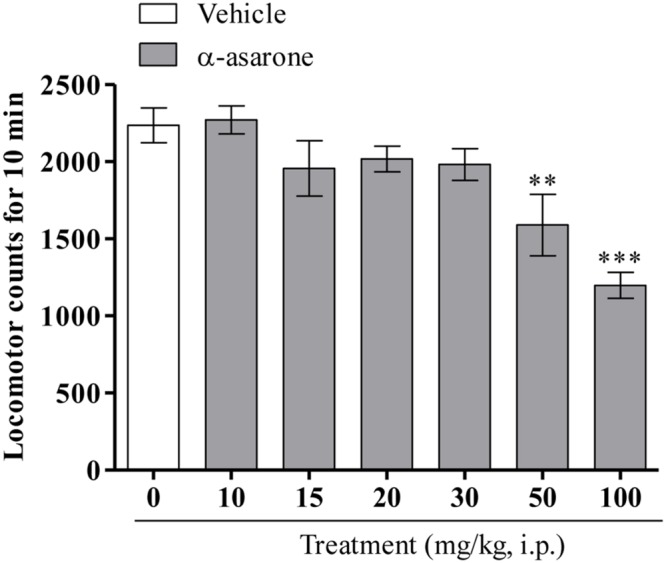
**Spontaneous locomotor activity.** Effect of the acute treatment of α-asarone (10, 15, 20, 30, 50, and 100 mg/kg, i.p.) *per se* on the spontaneous locomotor activity in mice. Values are expressed as mean ± SEM (*n* = 9–10). The statistical difference between vehicle control and α-asarone treated groups was analyzed using one-way ANOVA followed by *post hoc* Dunnett’s multiple comparison test. ^∗∗^*p* < 0.01 and ^∗∗∗^*p* < 0.001 as compared with the vehicle-control group.

As shown in (**Figure 3**), one-way ANOVA results revealed that α-asarone at lower doses (10, 15, 20, and 30 mg/kg, i.p.) did not significantly affect the spontaneous locomotor activity, whereas relatively higher doses of α-asarone (50 and 100 mg/kg, i.p.) significantly decreased locomotor activity when compared with the vehicle control group [*F*_(6,60)_ = 8.363, *p* < 0.0001].

### Involvement of the Noradrenergic System in the Antidepressant-Like Effect of α-Asarone

**FIGURE 4:**
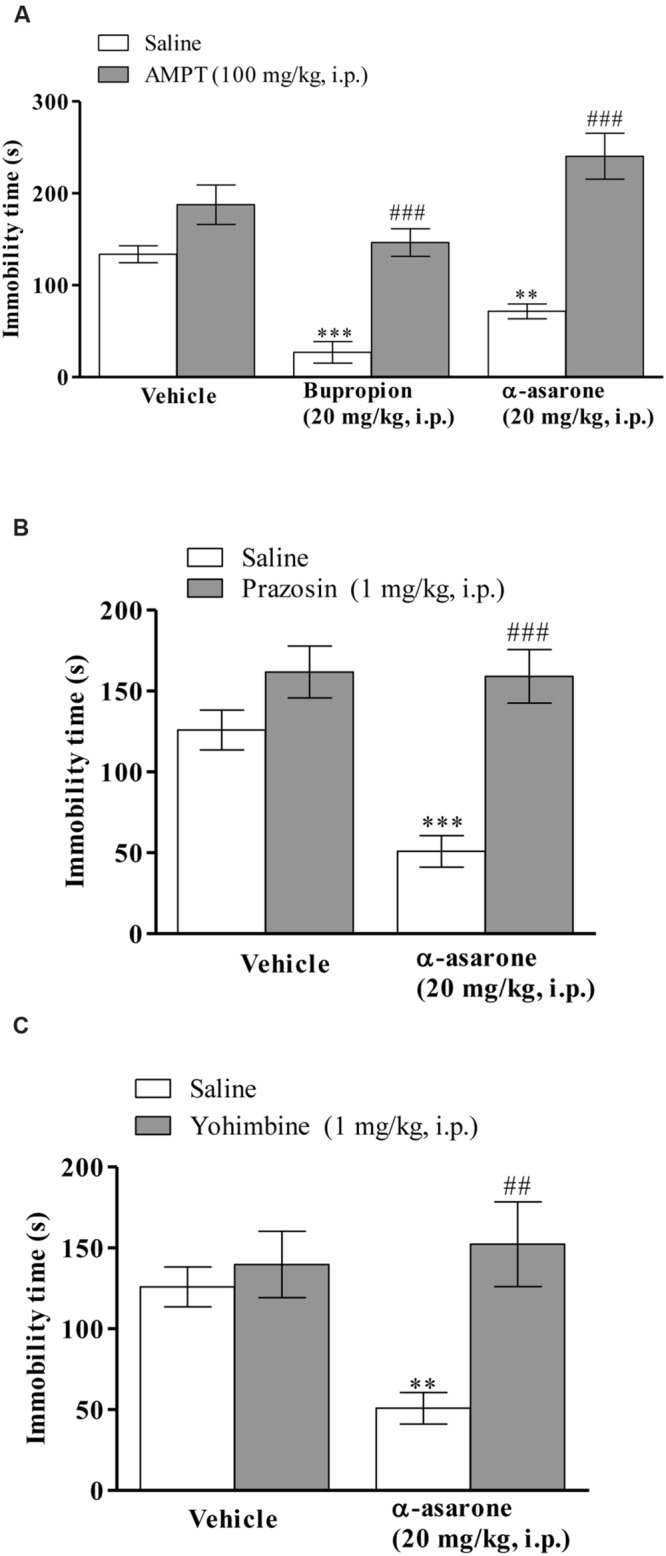
**Involvement of noradrenergic system in the antidepressant-like activity of α-asarone.** Effect of pre-treatment of mice with **(A)** AMPT (100 mg/kg, i.p., a catecholamine synthesis inhibitor) or **(B)** prazosin (1 mg/kg, i.p., an α_1_-adrenoceptor antagonist) or **(C)** yohimbine (1 mg/kg, i.p., an α_2_-adrenoceptor antagonist) on α-asarone (20 mg/kg, i.p.)- induced anti-immobility in the TST. Values are expressed as mean ± SEM (*n* = 8). The immobility time was analyzed using two-way ANOVA followed by *post hoc* Bonferroni test. ^∗∗^*p* < 0.01, and ^∗∗∗^*p* < 0.001 as compared with the vehicle group. ^##^*p* < 0.01 as compared with α-asarone (20 mg/kg, i.p.) *per se*, ^###^*p* < 0.001 as compared with the group treated with α-asarone (20 mg/kg, i.p.) or bupropion (20 mg/kg, i.p.) *per se*.

The results depicted in (**Figure 4A**) shows that the anti-immobility effect of α-asarone (20 mg/kg, i.p.) and positive control, bupropion (20 mg/kg, i.p.) was significantly blocked in AMPT (100 mg/kg, i.p.)-pretreated mice in the TST. Two-way ANOVA results revealed that there was a significant effect on AMPT pretreatment [*F*_(2,42)_ = 12.82, *p* < 0.0001], α-asarone or bupropion treatment [*F*_(1,42)_ = 72.92, *p* < 0.0001] and α-asarone or bupropion × AMPT interaction [*F*_(2,42)_ = 6.20, *p* < 0.0044]. Moreover, mice pretreated with prazosin (1 mg/kg, i.p.) significantly reversed the anti-immobility effect of α-asarone (20 mg/kg, i.p.) in the TST. Two-way ANOVA results showed a significant effect on prazosin pretreatment [*F*_(1,27)_ = 7.47, *p* < 0.0109], α-asarone treatment [*F*_(1,27)_ = 25.76, *p* < 0.0001] and α-asarone × prazosin interaction [*F*_(1,27)_ = 6.50, *p* < 0.0168; **Figure 4B**]. **Figure 4C** shows that yohimbine (1 mg/kg, i.p.) pretreatment significantly inhibited the anti-immobility effect of α-asarone (20 mg/kg, i.p.) in the TST. Two-way ANOVA results revealed a significant effect on α-asarone treatment [*F*_(1,26)_ = 9.41, *p* = 0.0050], α-asarone × yohimbine interaction [*F*_(1,26)_ = 5.43, *p* < 0.0278], but not with yohimbine pretreatment [*F*_(1,26)_ = 2.77, *p* = 0.1082].

### Involvement of the Serotonergic System in the Antidepressant-Like Effect of α-Asarone

**FIGURE 5:**
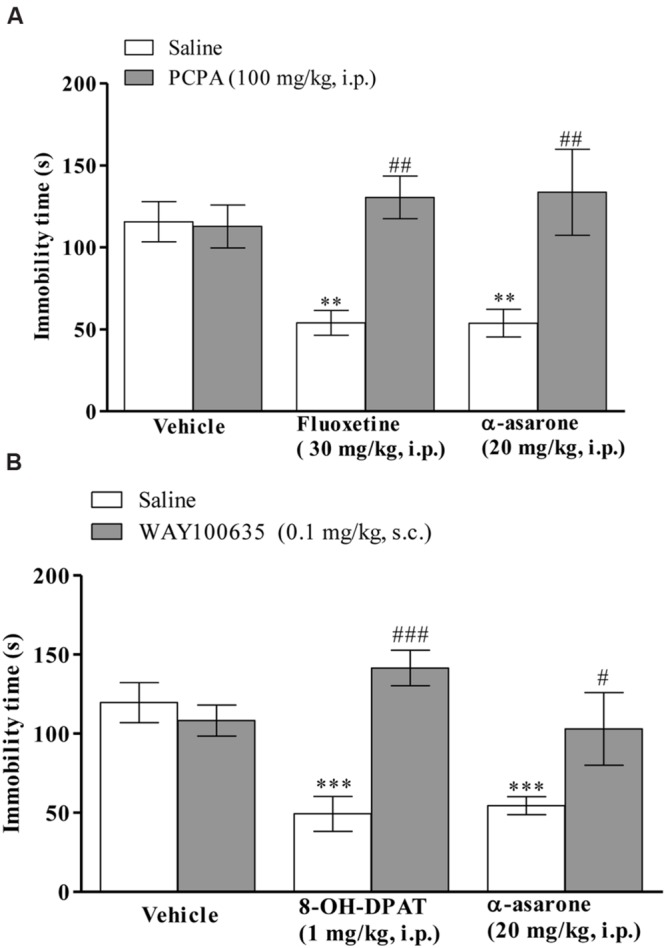
**Involvement of serotonergic system in the antidepressant-like activity of α-asarone.** Effect of pre-treatment with **(A)** PCPA (100 mg/kg, i.p., once daily for four consecutive days, a serotonin synthesis inhibitor) or **(B)** WAY100635 (0.1 mg/kg, s.c., a selective 5-HT_1A_ receptor antagonist) on α-asarone (20 mg/kg, i.p.)- induced anti-immobility in the TST. Values are expressed as mean ± SEM (*n* = 8). The immobility time was analyzed using two-way ANOVA followed by *post hoc* Bonferroni test. ^∗∗^*p* < 0.01 and ^∗∗∗^*p* < 0.001 as compared with the vehicle group. ^#^*p* < 0.05 as compared with the α-asarone (20 mg/kg, i.p.) *per se*, ^##^*p* < 0.01 as compared with the α-asarone (20 mg/kg, i.p.) or fluoxetine (30 mg/kg, i.p.) *per se* and ^###^*p* < 0.001 as compared with 8-OH-DPAT (1 mg/kg, i.p.) *per se*.

The anti-immobility effect of α-asarone (20 mg/kg, i.p.) or positive control, fluoxetine (30 mg/kg, i.p.) in the TST was prevented in PCPA (100 mg/kg, i.p., once daily for four consecutive days)-pretreated mice (**Figure 5A**). Two-way ANOVA results revealed that there was a significant effect of α-asarone or fluoxetine treatment [*F*_(1,42)_ = 17.98, *p* < 0.0001], α-asarone or fluoxetine × PCPA interaction [*F*_(2,42)_ = 5.02, *p* < 0.0111], and not with PCPA pretreatment [*F*_(2,42)_ = 1.38, *p* = 0.2630]. Besides that, mice pretreated with WAY100635 (0.1 mg/kg, s.c.) reversed the anti-immobility effect of α-asarone (20 mg/kg, i.p.) or 8-OH-DPAT (1 mg.kg, i.p.; **Figure 5B**). The two-way ANOVA results revealed a significant effect of WAY100635 pretreatment [*F*_(2,41)_ = 3.52, *p* = 0.0390], α-asarone or 8-OH-DPAT treatment [*F*_(1,41)_ = 15.45, *p* = 0.0003], α-asarone or 8-OH-DPAT × WAY100635 interaction [*F*_(2,41)_ = 7.44, *p* = 0.0018].

## Discussion

Depression is a heterogeneous affective disorder which particularly affects the mood and is associated with high rates of recurrence, relapses, and premature deaths. Globally, up to 20% of population are affected with major depression (Berton and Nestler, 2006). The TST is a well-validated and widely used animal model to screen potential antidepressants. It is an inexpensive, highly predictive, and is considered a high-throughput screening for the acute behavioral effects of antidepressants (O’Leary and Cryan, 2009).

The present TST results demonstrate that acute treatment of α-asarone at relatively lower doses (15 and 20 mg/kg, i.p.) showed an antidepressant-like effect in the mouse model of depression. This result is in accordance with the recent literature reported by Han et al. (2013) in which a significant antidepressant-like effect of α-asarone was demonstrated in both the FST and TST at doses of 10 and 20 mg/kg, i.p. However, α-asarone at relatively higher doses (50 and 100 mg/kg, i.p.) significantly increased the immobility time in the TST.

Furthermore, in order to eliminate false positive results of psychostimulant-like substances in the TST, the effect of α-asarone on spontaneous locomotor activity in mice was assessed. These results revealed that α-asarone (10–30 mg/kg, i.p.) did not affect the spontaneous locomotor activity, suggesting that the anti-immobility effect of α-asarone (15 and 20 mg/kg, i.p.) in the TST could not have been mediated through stimulation of the CNS. Moreover, α-asarone at higher doses (50 and 100 mg/kg, i.p.) significantly decreased the spontaneous locomotor activity and this result is in good agreement with the previous findings in which acute treatment of α-asarone (48, 50, and 100 mg/kg, i.p.) significantly decreased the locomotor activity in mice (Menon and Dandiya, 1967; Pages et al., 2010; Liu et al., 2012).

In addition, α-asarone (10–100 mg/kg, i.p.) did not affect the normal motor coordination as indicated in the horizontal wire test, thus alluding to absence of muscle relaxant property of α-asarone up to a dose of 100 mg/kg. This result corroborates with the previous report by Pages et al. (2010) which demonstrated that the motor coordination of mice was not affected when the mice were pretreated with α-asarone (22 and 60 mg/kg, i.p.) in the rotarod test. Therefore, it can be suggested that the increased immobility time in the TST, and a reduction in spontaneous locomotor activity at higher doses of α-asarone (50 and 100 mg/kg, i.p.) could be attributed to its depressant-like effect and not due to any muscle relaxant effect. The present study, however, could not clarify the exact mechanism(s) involved in the depressant-like effect of α-asarone at relatively higher doses. Based on our thorough literature search, it is postulated that GABAergic mediated mechanism could be involved in the depressant-like effect of α-asarone at higher doses. In earlier studies, α-asarone at relatively higher doses showed antiepileptic activity mediated through GABAergic mechanism in pentylenetetrazole or picrotoxin-induced seizures in mice (Pages et al., 2010). Similarly, the electrophysiological studies (Huang et al., 2013; Wang et al., 2014) also confirmed the facilitatory effect of α-asarone on GABA_A_ receptors.

The monoamine theory of depression was proposed by Schildkraut (1965) and states that depression is caused by functional deficit of the monoamine neurotransmitters mainly noradrenaline (NA) and/or serotonin. The currently available antidepressants which are very effective in treating major depression including monoamine oxidase inhibitors (MAOIs), tricyclic antidepressants (TCAs), selective serotonin reuptake inhibitors (SSRIs), noradrenaline reuptake inhibitors (NRIs) and serotonin and noradrenaline reuptake inhibitors (SNRIs). The key mechanism(s) of action of all of these drugs are similar, that is, by enhancing the brain’s noradrenergic and/or serotonergic transmissions (Berton and Nestler, 2006; Moret and Briley, 2011). Thus, the monoamine theory of depression is still considered a promising tool in the novel drug discovery for the treatment of major depressive disorder.

Down regulation of the central noradrenergic system and the ensuing reduction of brain noradrenaline level are the main key factors responsible for the pathophysiology of depressive disorder (Moret and Briley, 2011). AMPT is an inhibitor of tyrosine hydroxylase, a rate limiting enzyme in the biosynthesis of noradrenaline and dopamine (Widerlov and Lewander, 1978). Mice pretreated with AMPT showed a significant reduction in the brain noradrenaline and dopamine levels without affecting the levels of serotonin (Mayorga et al., 2001). It has also been reported that AMPT pretreated mice demolished the antidepressant activity of bupropion (a non-selective noradrenaline and dopamine reuptake inhibitor) in the TST (Kwon et al., 2010). Moreover, several studies elucidated the involvement of α_1_, and α_2_- adrenoceptors in the antidepressant-like effect of drugs in animal behavioral models of depression (Machado et al., 2009; Capra et al., 2010; Goncalves et al., 2012; Zeni et al., 2013). Evidence suggested that the brain α_1_-adrenoceptors were desensitized in the depressed patients and that the activation of α_1_- adrenoceptors restored the normal mood (Stone et al., 2003). In addition, chronic treatment with TCAs enhanced the density of α_1_- adrenoceptors (Stone et al., 2003) and importantly, the antidepressant effect of desipramine (a TCA) was blocked in the mice pretreated with prazosin (an α_1_-adrenoceptor antagonist) in the FST (Danysz et al., 1986). Furthermore, antagonism of presynaptic α_2_-adrenoceptors enhanced the noradrenaline levels and on the other hand, activation of post-synaptic α_2_-adrenoceptors facilitated the antidepressant-like effect (Zhang et al., 2009). Similarly, the antidepressant-like effect of clonidine (an α_2_-adrenoceptors agonist) was inhibited by yohimbine (α_2_-adrenoceptor antagonist) in the FST (O’Neill et al., 2001). It has also been found that the α_2_-adrenoceptors were up-regulated in depressed patients and that chronic antidepressant therapy decreased its up-regulation (Flügge et al., 2003). These evidences suggested the importance of the noradrenergic (α_1_ and α_2_ adrenoceptors) system for effective antidepressant therapy. In the present study, the mice pretreated with AMPT (a catecholamine synthesis inhibitor) prevented the antidepressant-like effect of α-asarone and in addition, the mice pretreated with prazosin (an α_1_-adrenoceptor antagonist) and yohimbine (α_2_-adrenoceptor antagonist) also abolished the antidepressant-like activity of α-asarone, indicating the involvement of the noradrenergic (α_1_ and α_2_ adrenoceptors) system in the antidepressant-like effect of α-asarone.

The down-regulation of brain serotonergic system is strongly implicated in depressive disorder (Mann, 2013). PCPA, a selective serotonin synthesis inhibitor, inhibits tryptophan hydroxylase and depletes serotonin level in the brain without affecting the brain noradrenaline and dopamine levels (Redrobe et al., 1998). Several studies highlighted that the pretreatment of PCPA inhibited the antidepressant-like effect of fluoxetine (a selective serotonin reuptake inhibitor) in the TST and FST (Machado et al., 2009; Kwon et al., 2010). The Positron emission tomography with [11C] WAY100635 in depressed patients revealed a decreased expression of 5-HT_1A_ receptors in several brain regions including frontal cortex and hippocampus (Drevets et al., 1999; Sargent et al., 2000). Furthermore, one of the mechanisms involved in the antidepressant-like effect of MAOI, TCAs, or SSRIs is mediated by its interaction with 5-HT_1A_ receptors (Hensler, 2002). In another study, it was found that the 5-HT_1A_ knockout mice treated with the fluoxetine or paroxetine (SSRIs) did not decrease the immobility time whereas desipramine (TCA) decreased the immobility in the TST (Mayorga et al., 2001), which clearly suggested the importance of 5-HT_1A_ receptors in the antidepressant effect of SSRIs.

In the present study, the mice pretreated with PCPA (a selective serotonin synthesis inhibitor) blocked the antidepressant-like effect of α-asarone. Furthermore, pretreatment of mice with WAY100635 (a 5-HT_1A_ antagonist) abolished the antidepressant-like effect of α-asarone. These results demonstrated the involvement of the serotonergic (particularly the 5-HT_1A_ receptors) system in the antidepressant-like effect of α-asarone.

In summary, our study demonstrate that acute treatment of α-asarone exhibited antidepressant-like activity at relatively lower doses (15 and 20 mg/kg, i.p.) without affecting either locomotor activity or motor coordination in mice. On the other hand, α-asarone at relatively higher doses (50 and 100 mg/kg, i.p.) significantly enhanced the immobility time in the TST and diminished the spontaneous locomotor activity which indicates a depressive-like effect of α-asarone at higher doses. Moreover, the antidepressant-like effect of α-asarone was prevented in the mice pretreated with AMPT or prazosin or yohimbine or PCPA or WAY100635, thereby indicating the involvement of both the noradrenergic and serotonergic systems in the antidepressant-like effect of α-asarone.

## Conclusion

This study results indicates that acute treatment of α-asarone exhibited a biphasic effect on the immobility time in the TST, with an antidepressant-like activity at lower doses and depressive-like effect at higher doses. The antidepressant-like effect of α-asarone is mediated by its interaction with noradrenergic (α_1_ and α_2_ adrenoceptors) and serotonergic (particularly, the 5-HT_1A_ receptors) systems. Therefore, we suggest that α-asarone could be singled out and further tested as a potential drug for the treatment of major depressive disorder.

## Author Contributions

RC designed, performed the experiments, analyzed the data, and wrote the manuscript, VP participated in the study design and critically revised the manuscript for important intellectual content. ZM critically revised the manuscript for important intellectual content. All authors read and approved the manuscript.

## Conflict of Interest Statement

The authors declare that the research was conducted in the absence of any commercial or financial relationships that could be construed as a potential conflict of interest.

## Abbreviations

CNS: central nervous system
GABA: gamma-aminobutyric acid
5-HT: 5- hydroxytryptamine
i.p.: intraperitoneal injection
s.c.: subcutaneous injection
TST: tail suspension test
